# Case Report: Meningoencephalitis With Thrombotic Occlusive Vasculopathy in a Young EBV-Naïve Boy Is Associated With a Novel *SH2D1A* Mutation

**DOI:** 10.3389/fimmu.2021.747738

**Published:** 2021-12-20

**Authors:** Jolanda Steininger, Raphael Rossmanith, Christoph B. Geier, Alexander Leiss-Piller, Lukas Thonhauser, Simone Weiss, Johannes A. Hainfellner, Michael Freilinger, Wolfgang M. Schmidt, Martha M. Eibl, Hermann M. Wolf

**Affiliations:** ^1^ Immunology Outpatient Clinic, Vienna, Austria; ^2^ Doctoral School Molecular Biology and Biochemistry, Institute of Molecular Biosciences, University of Graz, Graz, Austria; ^3^ Department of Pediatrics, Klinik Favoriten, Vienna, Austria; ^4^ Division of Neuropathology and Neurochemistry, Department of Neurology, Medical University of Vienna, Vienna, Austria; ^5^ Department of Pediatrics and Adolescent Medicine, Medical University of Vienna, Vienna, Austria; ^6^ Neuromuscular Research Department, Center for Anatomy and Cell Biology, Medical University of Vienna, Vienna, Austria; ^7^ Biomedizinische Forschungs GmbH, Vienna, Austria; ^8^ Sigmund Freud Private University- Medical School, Vienna, Austria

**Keywords:** CNS vasculopathy, meningoencephalitis, primary immunodeficiency, X-linked lymphoproliferative disease type 1, XLP1, multiple cerebral aneurysms, SH2D1A

## Abstract

X-linked lymphoproliferative disease (XLP1) is a combined immunodeficiency characterized by severe immune dysregulation caused by mutations in the *SH2D1A/SAP* gene. Loss or dysfunction of SH2D1A is associated with the inability in clearing Epstein-Barr-Virus (EBV) infections. Clinical manifestation is diverse and ranges from life-threatening hemophagocytic lymphohistiocytosis (HLH) and fulminant infectious mononucleosis (FIM) to lymphoma and antibody deficiency. Rare manifestations include aplastic anemia, chronic gastritis and vasculitis. Herein, we describe the case of a previously healthy eight-year old boy diagnosed with XLP1 presenting with acute non-EBV acute meningoencephalitis with thrombotic occlusive vasculopathy. The patient developed multiple cerebral aneurysms leading to repeated intracerebral hemorrhage and severe cerebral damage. Immunological examination was initiated after development of a susceptibility to infections with recurrent bronchitis and one episode of severe pneumonia and showed antibody deficiency with pronounced IgG1-3-4 subclass deficiency. We could identify a novel hemizygous *SH2D1A* point mutation affecting the start codon. Basal levels of SAP protein seemed to be detectable in CD8^+^ and CD4^+^ T- and CD56^+^ NK-cells of the patient what indicated an incomplete absence of SAP. In conclusion, we could demonstrate a novel *SH2D1A* mutation leading to deficient SAP protein expression and a rare clinical phenotype of non-EBV associated acute meningoencephalitis with thrombotic occlusive vasculopathy.

## Introduction

X-linked lymphoproliferative disease (XLP1) is a rare combined immunodeficiency characterized by severe immune dysregulation in response to viral infections, typically to Epstein-Barr virus (EBV) (1, 2). XLP1 is caused by mutations of *SH2D1A* ablating or functionally compromising SAP, an adaptor molecule that controls signaling downstream of several SLAM family transmembrane receptors expressed in NK-, NKT-, and T-cells (3). SAP is a 128-amino acid cytoplasmic adapter protein which consists of a 5-amino acid N-terminal sequence, an SH2 domain, and a 25-amino acid C-terminal tail (4). When activated, SAP mediates the expansion of activated T-cells during immune responses, induces the production of interferon-gamma (IFN-γ), and influences the functional profile of T-cell subsets (4). XLP1 presents with a dysfunction of clearing viral infections such as EBV, thus exposing XLP patients to EBV induced uncontrolled immune response, including severe and mostly fatal fulminant infectious mononucleosis and hemophagocytic lymphohistiocytosis. Approximately one third of the patients were reported to have lymphoproliferative disorders and one third were observed to have antibody deficiency (5, 6). Other rare but well-described clinical features include lymphoma, aplastic anemia, chronic gastritis, lymphomatoid granulomatosis and lymphocytic vasculitis (7–10). Due to diverse clinical symptoms and EBV-seronegativity in approximately 10% of the patients, early diagnosis of SH2D1A deficiency as the causative condition is challenging (5). Central nervous system (CNS) manifestations are a rare but severe complication of primary immunodeficiencies (PIDs) that range from predispositions to viral or bacterial infections of the CNS leading to meningitis or encephalitis (11–14), accumulation of toxic metabolites in the CNS (15, 16), CNS vasculitis (5, 17–23) as well as CNS involvement in hemophagocytic lymphohistiocytosis (24, 25). In XLP1 11 patients with CNS vasculitis have been described in the past (**STable 1**). These patients manifested with progressive cerebral lymphocytic vasculitis which is characterized by infiltration of CD8^+^ T-cells in blood vessels and vascular necrosis. One patient with limbic encephalitis with signs of CNS vasculitis has been described (26). Cerebral vasculitis is fatal in most of the XLP1 patients, despite the use of extensive immunosuppressive therapies (2). Early hematopoietic stem cell transplantation might be a treatment option leading to the regression of the vasculitis or CNS inflammation (27). In XLP1 the development of cerebral vasculitis or CNS inflammation can be independent of EBV infection. The antigenic stimulus leading to proliferative expansion of SH2D1A deficient T-cells in these patients is yet to be identified (8, 26–30). We herein report an eight-year-old male with a novel *SH2D1A* start codon mutation, drastically reducing SAP expression in peripheral T- and NK-cells, who presented with acute meningoencephalitis with thrombotic occlusive vasculopathy in the absence of EBV infection. Multiple cerebral aneurysms leading to repeated intracerebral hemorrhage and severe brain damage developed in the course of his disease.

## Case Report

**Figure 1:**
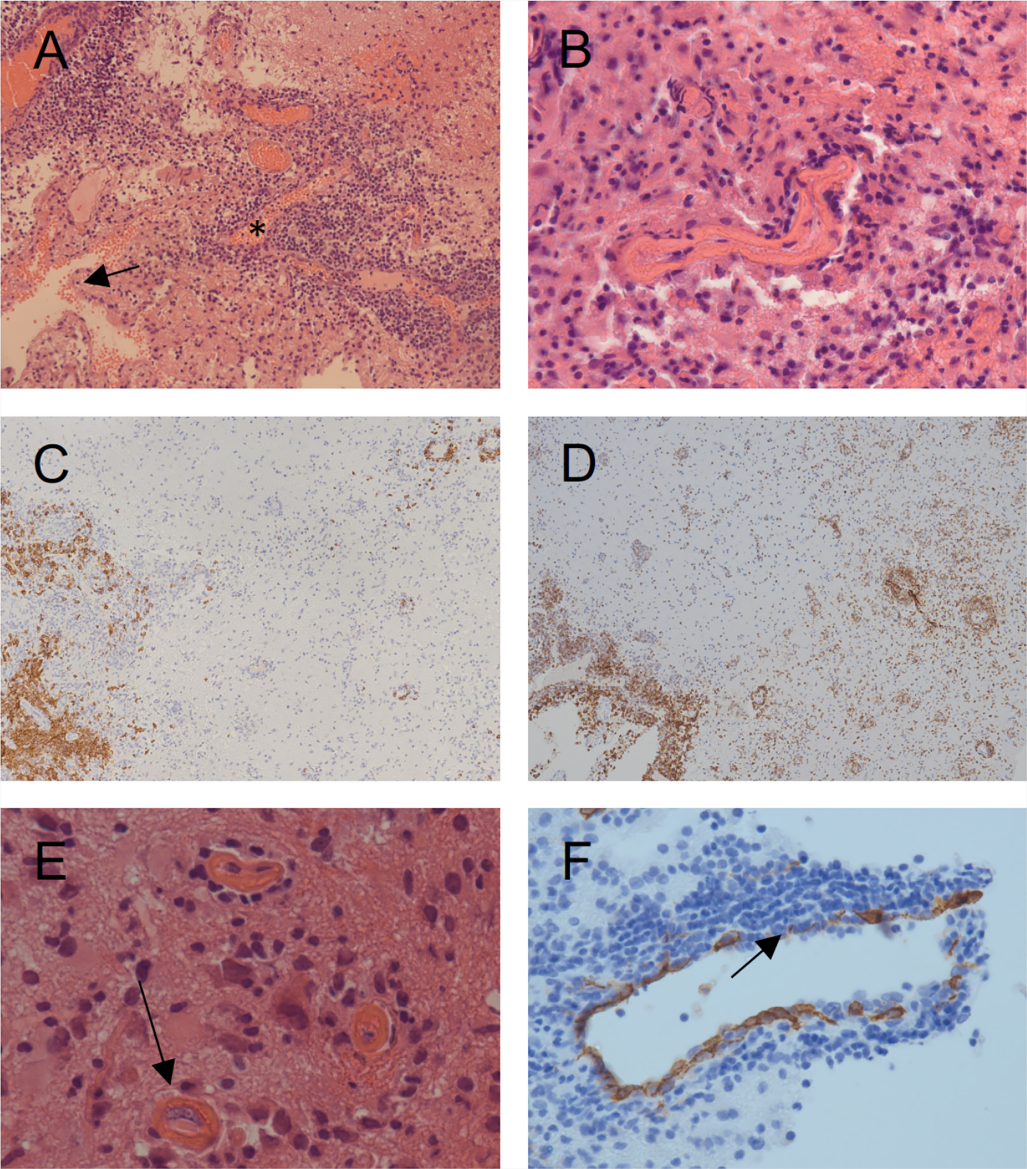
Histopathologic findings obtained by open surgery biopsy at age 8. **(A)** Hematoxylin and eosin (HE) staining showing diffuse lymphocytic infiltration involving the meninges (arrow), the vessels (asterisk) and the CNS tissue. **(B)** HE staining shows fibrinoid necrosis of the vessel walls with lymphocytic cell infiltrates. **(C)** Staining with a CD20 marker (brown) shows the distribution pattern of B-cells, primarily restricted to the meninges and the vessel walls. **(D)** Staining with a CD8 marker (brown) shows the distribution pattern of CD8^+^ T-cells with diffuse infiltration of the meninges, the vessel walls and the brain parenchyma. CD3^+^ T-cells showed a similar distribution pattern (data not shown). **(E)** Thrombotic occlusive vasculopathy (arrow) is shown. **(F)** CD34 staining of the vessel endothelia shows thin and fragmented endothelia (arrow) with partially thrombosed vessels, indicating severe vascular injury.

**Figure 2:**
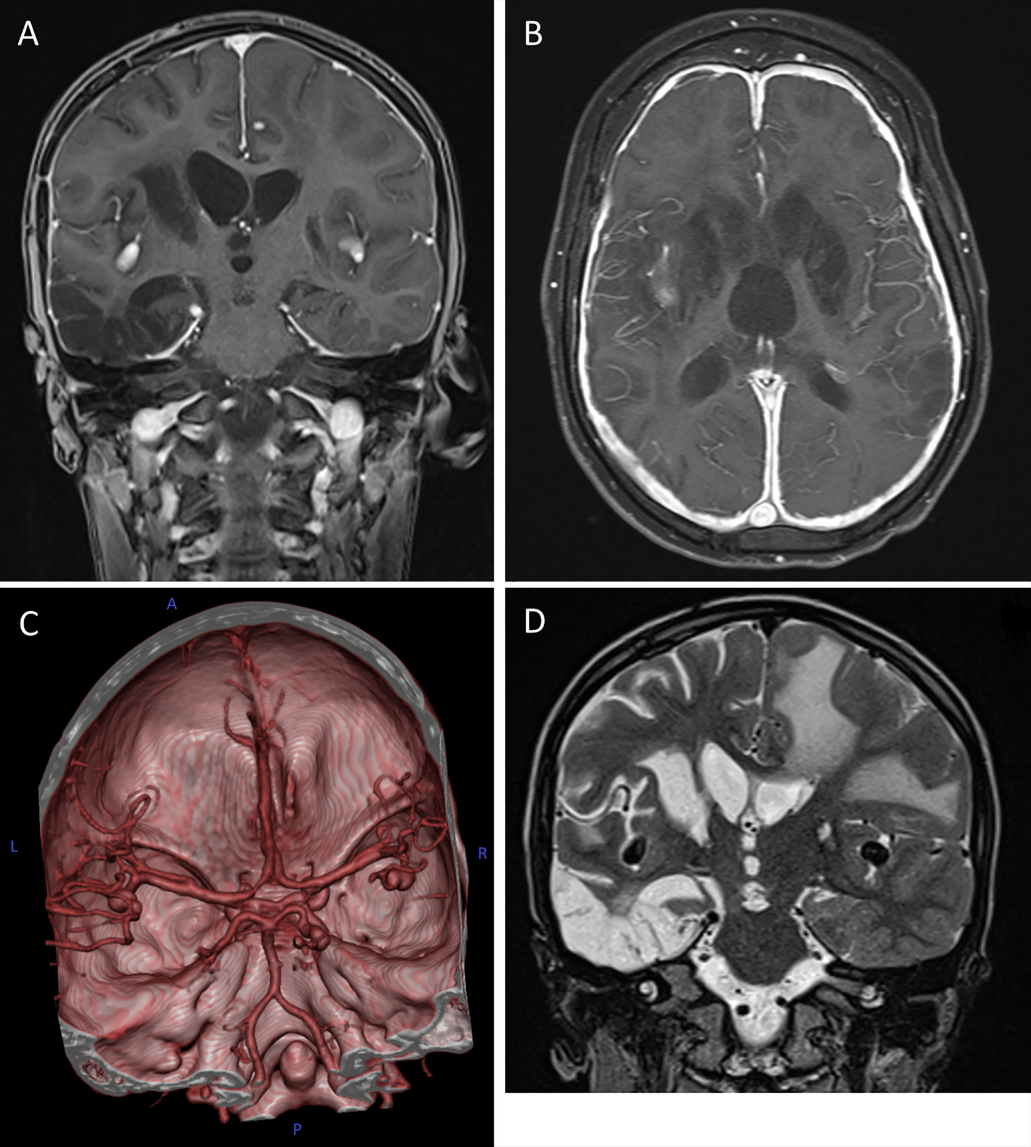
Cerebral magnetic resonance imaging (cMRI) of the index patient at age 10. **(A, B)** T1-weighted, contrast enhanced MRI scans in coronal **(A)** and axial **(B)** plane depicting the hypertrophic pachymeningitis with bilateral basal ganglia, midbrain and cortical lesions. Note the postinfectious internal hydrocephalus. **(C)** 3D reconstitution of the vascular system of the brain showing bilateral media aneurysms and a posterior cerebral artery aneurysm on the right without signs of vessel narrowing. **(D)** T2-weighted MRI scan in coronal plane highlighting the extensive frontotemporal white matter edema on the left, global parenchymal atrophy and the medial cerebral artery aneurysms bilaterally.

**Figure 3:**
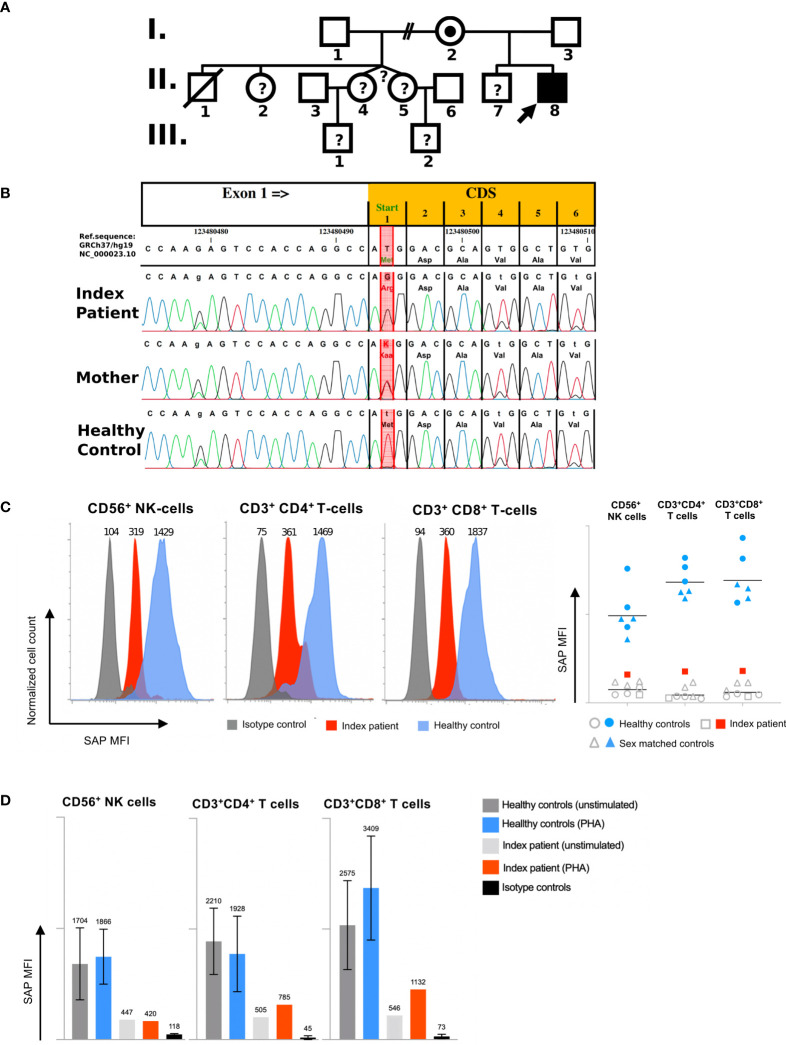
Molecular and functional characterization of a novel *SH2D1A* mutation: **(A)** Family Pedigree. The arrow indicates the index patient. Mutation analysis confirmed a hemizygous *SH2D1A* mutation in patient II,8 and female carrier status in his mother I,2. One half-brother died at the age of 3 from acute leukaemia after suspected EBV infection (II,1), without confirmation of the XLP1 genotype by sequencing. No genetic analysis could be performed in other family members (indicated by question marks). **(B)** Results of Sanger sequencing of *SH2D1A* of the index patient, the mother and a healthy male individual are shown. Red column indicates position of the start codon mutation (ChrX:g.123480494 T>G; GRCh37/hg19). **(C)** Intracellular SAP expression due to novel *SH2D1A* mutation. Mean fluorescence intensities (MFI) representing intracellular SAP expression in CD56^+^ NK-cells, CD4^+^ T-cells, and CD8^+^ T-cells of one representative healthy control (blue), and the index patient (red) are shown in histograms (isotype control staining is shown in grey). Diagram of MFI representing SAP expressions of six healthy controls consisting of 3 male sex matched controls (shown as blue triangles) and 3 anonymous blood donors (shown as blue dots), the index patient (shown as red squares) and relating isotype control staining (shown as grey rings, triangles and squares) are shown in the right figure (lines represent geometric mean). **(D)** Quantification analysis of intracellular SH2D1A expression. MFI of intracellular SH2D1A expression in CD56^+^ NK-cells, CD3^+^CD4^+^ T-cells and CD3^+^CD8^+^ T-cells from five healthy controls including 3 male sex matched controls and 2 anonymous blood donors as well as the SH2D1A-deficient index patient following 3 days of PHA (phytohemagglutinin)-stimulation are shown (bars and top numbers represent mean values, error indicators represent standard deviations, staining of isotype controls are shown in black).

The patient was the second child born to non-consanguineous healthy parents of Caucasian origin. Past medical history revealed recurrent mild upper respiratory infections and one episode of pneumonia at the age of 3. The patient presented at 8 years of age with a one-month history of recurrent headaches and behavioral changes. Subsequent cranial magnetic resonance imaging (cMRI) revealed multiple bilateral necrotizing lesions in the basal ganglia as well as in the right temporal lobe. Protein levels in cerebrospinal fluid (CSF) were elevated at 941mg/dL with increased cell count (100 cells/µL) and lymphocytic pleocytosis. *Human herpesvirus 7* (HHV-7) PCR was tested positive in the CSF as well as in peripheral blood, while PCR for *Epstein–Barr virus* (EBV), *Cytomegalovirus*, *Herpes simplex virus*, *Human herpesvirus 6* and enterovirus were negative. Bacterial cultures of the CSF and peripheral blood, including *Borrelia* and *Mycoplasma*, were negative. Additionally, an extensive autoimmunologic work-up, including antineuronal antibody testing (e.g. NMDAR, AMPAR, GABA(B)R, LGI1 and CASPR2 antibodies) was unsuspicious. Open cerebral biopsy showed extensive lymphomonocytic infiltration of the meninges and parenchyma as well as most perivascular regions with fibrinoid necrosis of the vessel walls and brain parenchyma (**Figure 1**). EBV *in situ* hybridization and HHV-7 PCR of the tissue was negative. Overall, these findings indicated a florid meningoencephalitis with thrombotic occlusive vasculopathy of unknown cause, although HHV-7 infection as a possible trigger could not be excluded. The patient was treated with broad-spectrum antibiotics, acyclovir, as well as an aggressive immunosuppressive therapy with mycophenolate mofetil (MMF) and high-dose glucocorticoids which led to clinical improvement. Long-term immunosuppressive therapy was continued after the discharge. After 3 months the patient was readmitted with acute left-side hemiparesis due to intraparenchymal and ventricular hemorrhage. After trepanation and decompression, the hemiparesis clinically improved, while the patient developed organic brain disorder and a central diabetes insipidus, which was treated with vasopressin analogs and antipsychotics. At the age of 10, neurological functions rapidly declined with changes in movement patterns and increased myotonus of the lower extremities. A cMRI revealed a hypertrophic pachymeningitis with progression of the lesions bilaterally within the hemispheres of the brain with mild hemorrhage (**Figure 2**). Additionally, fusiform aneurysms were found within the M2 and M3 segments of the right middle cerebral artery and the right posterior cerebral artery without signs of vessel narrowing. Protein levels in CSF were elevated, with increased cell count at 25 cells/µL. We could detect immunoglobulins within the CSF, without oligoclonal bands. HHV-7 PCR was again tested positive in CSF and peripheral blood. Upon escalation of immunosuppressive therapy with glucocorticoids and MMF as well as short-term intravenous immunoglobulin therapy, the patient clinically improved. Two months later, the patient was readmitted for the third time with acute sepsis due to lobar pneumonia and transferred to the intensive care unit (ICU), where he survived resuscitation following sudden cardiac arrest. A subsequent cranial computer tomography scan showed massive intraventricular hemorrhage, which was treated with an external ventricular drain (EVD), however extensive neurological dysfunction developed. The patient received antibiotic therapy and supportive care as well as high dosage glucocorticoids and MMF. During recovery at the ICU the patient developed the reoccurrence of a massive intracerebral hemorrhage after removal of EVD which resulted in progression of extensive neurological dysfunctions. The patient entered a minimally conscious state with flaccid tetraparesis, lost protective reflexes and developed vegetative instability. He required surgical placement of a tracheostoma and the insertion of a PEG (percutaneous endoscopic gastrostomy) tube. In the following year, the patient developed recurring respiratory infections as well as septic pyelonephritis, requiring repeated intravenous antibiotic therapy. Necrotizing CNS inflammatory disease in combination with susceptibility to infections lead to evaluation for PID. Extensive immunological work-up revealed severe IgG1-IgG3-IgG4 subclass deficiency (**Table 1**). Antibody response to previous vaccination against tetanus and diphtheria, T-cell dependent protein antigens, was absent. T-cell independent polysaccharide vaccine against *Pneumococcus* resulted in IgM and IgA antibody response but defective IgG- and IgG2-response (**Table 1**). Repeated serum EBV serology was negative. We could detect reduced switched memory B-cells (IgD^-^CD27^+^ cells) and complete absence of iNKT-cells (CD3^+^Vα24^+^Vβ11^+^) previously described in XLP1 (**Table 1**). Long-term immunoglobulin replacement therapy was initiated. Family history revealed a half-brother from the same mother with a previous partner who died in early childhood at 3,5 years of age from fulminant leukemia many years before the patient was born. The definite genotype as well as EBV status of the half-brother is unknown. The family pedigree of the patient is shown in **Figure 3A**. A T to G transposition within the start codon of the *SH2D1A* gene (ChrX:g.123480494 T>G, NM_002351.5:c.2T>G, NP_002342.1:p.M1?, Exon 1) was revealed independently by targeted resequencing of genes associated with PID’s and whole exome sequencing. Sequencing of the patient’s mother confirmed maternal segregation of the variant (**Figure 3B**). We could not identify any other pathogenic or variants of unknown significance in PID genes nor those associated with vasculopathies or autoimmunity. We hypothesized that this novel variant resulted in an aberrant protein expression of SH2D1A. SAP protein expression in the patient’s peripheral blood mononuclear cells (PBMCs) was evaluated using flow cytometry. We could demonstrate a significant reduction of intracellular SAP-intensities within NK-cells, CD4^+^ T-cells and CD8^+^ T-cells of the patient as compared to those of six healthy controls (3 anonymous blood donors and 3 sex-matched controls) (**Figure 3C**). The findings indicate that the start codon mutation led to a drastic reduction but possibly not a complete absence of SH2D1A expression. Additionally, we stimulated PBMCs, isolated from the patient and five healthy controls (2 anonymous blood donors and 3 sex-matched controls), with phytohemagglutinin (PHA) for 3 days. We could demonstrate, that SH2D1A expression in the CD4^+^ T-cells and CD8^+^ T-cells of the patient seemed to be upregulated following PHA stimulation but was still drastically decreased compared to detected SAP levels in cells of healthy controls (**Figure 3D**). Therefore, the results indicate the reduced translational activity for SAP protein expression in the patient. The final diagnosis of XLP1 with EBV-negative CNS inflammation was established. Nevertheless, the patient’s clinical condition, despite neurological rehabilitation and extensive therapeutic efforts, did not improve considerably, with indication of neurological irreversibility. Therefore, bone marrow transplantation was not considered as a therapeutic option. The patient is under continuous immunosuppressive as well as immunoglobulin replacement therapy and receives supportive care. Two years later the patient developed pulmonal vasculitis and is up to this point hospitalized under continuous supportive care.

**Table 1:** Immunological characterization.

Antibody Serology				
**age of the patient**	**normal range (age-matched)**	**11 years**	**12 years**	13 years*
				
**IgG (mg/dL)**	790-1700	470*L	998	899
**IgG1 (mg/dL)**	500-880	122*L	172*L	374*L
**IgG2 (mg/dL)**	150-600	321	956*H	622*H
**IgG3 (mg/dL)**	20-100	14*L	18*L	14*L
**IgG4 (mg/dL)**	8-120	<6*L	24	6*L
**IgM (mg/dL)**	90-350	218	189	218
**IgA (mg/dL)**	76-450	427	673*H	427
**IgE (mg/dL)**	≤100	2.79	3.8	2.79

**serum antibody titer before and after booster vaccination**				
**T-dependent protein antigens**	**normal range++) (number of age-matched controls)**	**before vaccination**	**after vaccination+)**	
tetanus toxoid IgG (IU/ml))	1.67-12.14 (33)	0.16*L	0.86*L	
diphtheria toxoid lgG (IU/ml)	0.42-7.22 (22)	0.01*L	0.03*L	
**T-independent polsaccharide antigen**				
23-valent pneumococcal polysaccharide IgG (reciprocal titer) antibody response	428-10785 (41)	77*L	180*L	
23-valent pneumococcal polysaccharide IgM (reciprocal titer) antibody response	164-11943 (41)	768	1335	
23-valent pneumococcal polysaccharide IgA (reciprocal titer) antibody response	347-3452 (23)	174*L	489	
23-valent pneumococcal polysaccharide IgG2 (reciprocal titer) antibody response	143-2326 (23)	66*L	90*L	
23-valent pneumococcal polysaccharide (ratio IgG/lgM antibody response	0.17-45.9 (41)	0.1*L	0.135*L	

**EBV serology**				
**age of the patient**	**11 years**	**12 years**	13 years*	
EBV EA IgG antibody	neg	neg	neg	
EBV CA IgG antibody	neg	neg	pos	
EBV CA IgM antibody	neg	neg	neg	
EBV EBNA antibody	neg	neg	pos	

**Immunological phenotype**				
**age of the patient**	**normal-range (age-matched)**	**12 years**	13 years*	
			
leukocytes cells/μL	4500-13500	4180*L	10880	
CD3^+^ cells/μL (% of lymphocytes)	694-2976 (53-85%)	1718 (70.88%)	1479 (71.53%)	
CD4^+^ cells/μL (% of lymphocytes)	386-2022 (31-66%)	942 (38.85%)	836 (40.42%)	
CD8^+^ cells/μL (% of lymphocytes)	297-1011 (21-43%)	995 (41.04 %)	767 (37.10%)	
CD56^+^ cells/μL (% of lymphocytes)	98-680 (6-29%)	390 (16.07%)	242 (11.72%)	
CD19^+^ cells/μL (% of lymphocytes)	71-549 (7-23%)	305 (12.57%)	300 (14.50%)	
IgD^-^ CD27^+^ (% of CD19^+^)	7.81-27.45%	4.29%*L	2.49%*L	
IgD^+^ CD27^+^ (% of CD19^+^)	6.81-30.53%	10.23%	7.31%	
IgD^+^ CD27^-^ (% of CD19^+^)	45.84-80.36%	84.85%*H	89.77%*H	
CD62L^+^ 45RA^+^ (% of CD4^+^)	34.52-65.51%	17.49%*L	87.81%*H	
CD62L^+^ 45RA^-^ (% of CD4^+^)	19.32-37.24%	0.02%*L	none detected	
CD62L^-^ 45RA^-^ (% of CD4^+^)	9.21-25.87%	0.02%*L	0.04%*L	
CD62L^-^ 45RA^+^ (% of CD4^+^)	1.46-12.26%	82.45%*H	12.5%*H	
CD62L^+^ 45RA^+^ (% of CD8^+^)	15.55-63.15%	17.55%	71.02%*H	
CD62L^+^ 45RA^-^ (% of CD8^+^)	1 .84-9.51 %	0.53%*L	0.09%*L	
CD62L^-^ 45RA^-^ (% of CD8^+^)	2.77-25.25%	0.05%*L	none detected	
CD62L^-^ 45RA^+^ (% of CD8^+^)	22-65.67%	81.87%*H	28.9%	
iNKT cells (% of CD3^+^)	0.01-1%	none detected	none detected	

Immunological laboratory parameters of the XLP1 patient compared to an age matched healthy control cohort. *L and *H indicate values below and above the age-matched normal range. +) antibody response in the patient examined eight weeks after booster vaccination; ++) antibody response in healthy controls examined 6-8 weeks after booster vaccination. *under IVIG substitution.

## Discussion

Since the first report by Purtilo et al., describing the key features of XLP1 and linking the disease to a dysfunction of clearing EBV, rare clinical manifestations even in the absence of EBV exposure have been described in about 10% of the patients (31). This suggests that development of immune dysregulation in these patients might be independent of EBV infection (5, 7, 32–34). While XLP1 is primarily described as being associated to EBV infections in the majority of cases, EBV-seronegativity might hamper early diagnosis of SH2D1A deficiency. We herein report an XLP1 patient, harboring a novel *SH2D1A* mutation, who presented with EBV-negative meningoencephalitis with thrombotic occlusive vasculopathy at 8 years of age. We could identify a start codon variant in the *SH2D1A* gene. This variant represents a novel mutation which was predicted to reduce the efficiency of the start codon (35, 36). Consequently, intracellular flow-cytometry showed a drastic reduction of SH2D1A protein in the patients CD4^+^ and CD8^+^ T-cells as well as NK-cells but indicated residual protein expression at the same time. This might be caused by a low translational activity. Nevertheless, the drastic reduction of functional SH2D1A resulted in a severe form of XLP1. The remaining SAP protein expression could be due to minor but not a complete absence of translation initiation activity of the mutated AGG codon. Still, this is unlikely, since the exchange of purine and uridine bases in the second position of the AUG start codon are known to destabilize codon-anticodon base pairing (35, 37). Alternatively, the usage of an upstream or downstream cryptic translation initiation site could play a role (35, 38). Although no other in-frame AUG codon is present in the reference transcript (NM_002351.5), several possible non-AUG (cryptic) in frame translation initiation sites in a Kozak-like sequence environment can be found.

The patient’s half-brother, who died of lymphoma at 3,5 years of age likely carried the same mutation. Our patient presented with acute and severe CNS inflammation and hypogammaglobulinemia was detected two years after the presentation with neurological deficiencies. EBV seronegativity was shown in several independent analyses, even though interpretation of the results is ambiguous due to the antibody deficiency of the patient. Although we cannot entirely exclude a previous contact to EBV, EBV PCR of the tissue, the CSF and the blood was negative throughout the active cerebral inflammation. The patient never showed clinical symptoms of EBV infection and we could not demonstrate a sudden loss of B-cells, typical for EBV infections (39). The absence of NKT-cells in our patient is similar to other reported XLP1 patients in the literature (40). To our knowledge, 11 XLP1 patients, who suffered predominant from CNS manifestations have been described so far (**STable 1**). These manifestations included cerebral vasculitis and encephalitis. Comparable to our patient, 7 of these patients presented in the absence of EBV infections, as was defined by negative EBV PCR of the blood, the brain biopsy or the CSF (8, 26–30). The clinical history of these patients was dominated by the development of a progressive and in the majority of cases fatal vasculitis, presumably caused by uncontrolled proliferation and activation of CD8^+^ T-cells, which resulted in aneurysm formation, consecutive hemorrhages and finally intracerebral damages. The pathogenesis of CNS vasculitis in SAP-deficient patients is still unknown and the molecular trigger responsible for the activation of the uncontrolled T-cell response in previously described cases were rarely identified. Although CNS vasculitis in XLP1 patients was first associated with active EBV infection, more recent publications showed evidence of EBV-independent mechanisms (27, 30). Still, the mechanism that drives the inflammation remains elusive. HHV-7 was the only conspicuous infectious trigger that might play a role in the development of autoimmune CNS inflammation in our patient. HHV-7 was repeatedly detected in the patient’s CSF using PCR, even though HHV-7 PCR of the tissue was negative. HHV-7 antibody titers were not measured and would be of little value given the antibody deficiency of the patient. Therefore, primary infection and reactivation could not be differentiated (41). CNS involvement following primary HHV-7 infection is rare in children but more common when primary infection is delayed into adolescence (42). HHV-7 could function as a potential driver of autoinflammation since it establishes a life-long latency by initiating numerous immune evasion strategies such as upregulation of TNF‐α, TGF‐β and IFN‐γ as well as decrease of IL‐2, mitogen‐ and cytokine‐induced cellular proliferative responses (43). In 2015, Gray et al. presented an XLP1 patient, that manifested with a cerebral vasculitis that was also associated with HHV-7 positivity in the CSF (27). They linked the failure of clearing the HHV-7 infection to the consecutive lympho-proliferation with cerebral vasculitis. Our case report underlines the possibility of non-EBV associated CNS inflammation as a rare clinical manifestation in patients harboring a *SH2D1A* mutation. Our patient was a unique case of XLP1 manifesting with meningoencephalitis combined with thrombotic occlusive vasculopathy. Although not proven beyond doubt by histology, vasculitis can likely be the cause of this vasculopathy. In addition, our findings might support the role of HHV-7 as a possible trigger in the pathogenesis of cerebral vasculitis. Recent findings indicate that various forms of PIDs are associated with CNS manifestations and vasculitis (17). We therefore suggest that children with unexplained autoimmune CNS inflammation as a primary clinical manifestation should be investigated for an underlying primary immunodeficiency.

## Data Availability Statement

The original contributions presented in the study are included in the article/**Supplementary Material**. Further inquiries can be directed to the corresponding author.

## Ethics Statement

Ethical review and approval was not required for the study on human participants in accordance with the local legislation and institutional requirements. Written informed consent to participate in this study was provided by the participants’ legal guardian/next of kin. Written informed consent was obtained from the minor(s)’ legal guardian/next of kin for the publication of any potentially identifiable images or data included in this article.

## Author Contributions

JS, RR, and CBG analyzed and interpreted the results, created the figures and tables and actively wrote the manuscript. AL-P performed the genetic testing evaluation. CBG and RR conducted and interpreted measurements of functional assays in T-cells. Critical revision of the manuscript was done by LT, SW, CBG, JAH, MF, WMS, and HMW, who also cared for the patient and were actively involved in our research investigation. MME interpreted and analyzed results. HMW took overall responsibility for the research performed in this study and guided the writing of the manuscript. All authors have read and approved the contents of the manuscript and are accountable for all aspects of the work.

## Conflict of Interest

Author MME is employed by Biomedizinische Forschungs GmbH, Vienna, Austria.

The remaining authors declare that the research was conducted in the absence of any commercial or financial relationships that could be construed as a potential conflict of interest.

## Publisher’s Note

All claims expressed in this article are solely those of the authors and do not necessarily represent those of their affiliated organizations, or those of the publisher, the editors and the reviewers. Any product that may be evaluated in this article, or claim that may be made by its manufacturer, is not guaranteed or endorsed by the publisher.

## Supplementary Material

Supplementary Table 1 Clinical information about 11 XLP 1 patients described in the literature presenting with cerebral vasculitis, YA, years of age; MA, months of age; CSF, cerebral spinal fluid; BM, bone marrow.
