# Supplementary material for: Case Report: Meningoencephalitis With Thrombotic Occlusive Vasculopathy in a Young EBV-Naïve Boy Is Associated With a Novel SH2D1A Mutation
Source: Front Immunol. 2021 Dec 20;12:747738. doi: 10.3389/fimmu.2021.747738 (PMC8721048; doi:10.3389/fimmu.2021.747738)
Supplement: Supplementary Table 1 — Clinical information about 11 XLP 1 patients described in the literature presenting with cerebral vasculitis, YA, years of age; MA, months of age; CSF, cerebral spinal fluid; BM, bone marrow. [file Table_1.docx]

| **PMID**  **(year of publication)** |  | **Other manifestations** | **Age of diagnosis of XLP** | **Age of manifestation of vasculitis/CNS involvement** | **EBV status** | **Immunologic data** | **Histology** | **HSCT** | **Outcome** | **Mutation** |
| --- | --- | --- | --- | --- | --- | --- | --- | --- | --- | --- |
| 2986573 (1985) |  | - | 8 YA | 8 YA | EBV serology negative  EBV genome positive (DNA hybridization) | Hypogammaglobulinemia | Not reported | No | Lethal | Not reported |
| 8959062 (1996) |  | Cutaneous vasculitis | 17 YA | 17 YA | EBV serology negative  EBV genome positive in tissue (testing strategy unknown) | Hypogammaglobulinemia | Intense lymphocytic infiltrate | No | Unknown | Not reported |
| 11133747 (2001) |  | Virus- associated hemophagocytic syndrome (13 MA),  Chorioretinitis,  Chronic lung disease (Bronchiectasis) | 12 YA | 12 YA | EBV PCR (CSF) negative,  EBV in stu hybridizaton negative (brain tissue)  EBV PCR (brain tissue) positive  EBV serology (VCA) positive  (EBNA not performed) | Absolute and relative CD8^+^T-cell pleocytosis  inverted CD4^+^/CD8^+^ T-cell ratio  Hypogammaglobulinemia (IgG subclass) | Vessel infiltrates consisted primarily of CD8+ T cells (Immunohistochemistry),  Widespread microscopic necrotizing arteritis that resembled polyarteritis nodosa (CNS, heart, kidneys, testes, pancreas, eye & peripheral nerves) | No | Lethal | c.164C>T |
| 15682426 (2005) |  | Infectious mononucleosis (EBV induced) | 22 YA | 22 YA | EBV PCR (blood) negative | Hypogammaglobulinemia, Leucopenia, Neutropenia, Reduced B cells | Perivascular infiltrates of CD8+ T-cells of the lung, skin & brain | No | Unknown | c.321C>G |
| 16611784 (2006) |  | Burkitt lymphoma (7 YA),  Diffuse large B-cell immunoblastic lymphoma (18 YA),  Susceptibility to respiratory infections | 18 YA | 18 YA | EBV PCR (blood) positive  EBV PCR (CSF) positive | Not reported | No normal brain tissue available for histologic evaluation of blood vessels to confirm vasculitis (due to CNS lymphoma) | Yes | Lethal | Deletion exon 1 |
| 17620557 (2007) |  | B-cell non-Hodgkin lymphoma (monoclonal kappa, EBV negative, 9 YA) | 16 YA | 16 YA | EBV PCR (CSF) negative  EBV in situ hybridization (brain tissue) negative | Hypogammaglobulinemia (reduced IgG, IgM and IgA) | Vasculitis with infiltration of T cells (mainly CD8+ T-cells), granulomata,  absence of plasma cells | No | Lethal | None found |
| 19621458  (2009) | Patient 1 | - | 18 YA | 18 YA | EBV PCR (brain tissue, CSF, blood and BM) negative  EBV serology negative | Normal IgG, elevated IgM and reduced IgA  γδT-cell clone found | Vascular and parenchymal infiltration by CD8+ T-cells with necrosis of vessel walls | No | Lethal | c.163C>T |
|  | Patient 2 | Upper respiratory infection, Complicated  hereditary spherocytosis | 32 YA | 31 YA | EBV PCR (BM, CSF and blood) negative.  EBV serology negative | Eosinophilia, reduced B-cells, Ig levels were normal  γδT-cell clone found | Lymphocytic vasculitis with involvement of vessels in the brain, lung, liver & heart | No | Lethal | c.163C>T |
| 23816555 (2013) |  | Burkitt lymphoma (EBV negative, 3YA) | 5 YA | 5 YA | EBV PCR neg | Not reported | Not reported | No | Vegetative | c.163C>T |
| ﻿26433589 (2015) |  | Lymphocytic synovitis  with a mixture of CD4+ and CD8+ T-cells (5 YA),  Transient aplastic anaemia,  Chronic active pangastritis (CD8+ infiltrates | 9 YA | 9 YA | EBV PCR (CSF, blood) negative,  HHV-7 PCR from CSF positive | Low IgG, high IgA and IgM, normal T-cell numbers, normal CD4:CD8 ratio, elevated peripheral blood B-cells, almost absent NKT-cells, reduced antibody response to vaccines  No γδT-cell clone found | Not reported | Yes | Clinical remission of vasculitis | c.96G>C |
| 30138256 (2019) |  | Burkitt lymphoma (EBV negative, 14 YA) | 14 YA | 14 YA | EBV PCR (blood, CSF, brain tissue) negative  EBV serology: past exposure (VCAIgM  negative, VCA-IgG positive, negative EBNA). | Hypogammaglobulinemia,  reduced class-switched memory B-cells, lack of NKT cells  No γδT-cell clone found | Perivascular invasion by  CD8^+^ T-cells, no B-cells | No | Lethal | c.201G>A |
